# Septic arthritis of the knee: clinical and laboratory comparison of groups with different etiologies

**DOI:** 10.6061/clinics/2016(12)07

**Published:** 2016-12

**Authors:** Camilo Partezani Helito, Paulo Renan Lima Teixeira, Priscila Rosalba de Oliveira, Vladimir Cordeiro de Carvalho, José Ricardo Pécora, Gilberto Luis Camanho, Marco Kawamura Demange, Ana Lucia Munhoz Lima

**Affiliations:** I Faculdade de Medicina da Universidade de São Paulo, Departamento de Ortopedia e Traumatologia, Divisão de Cirurgia do Joelho, São Paulo/, SP, Brazil; II Faculdade de Medicina da Universidade de São Paulo, Departamento de Ortopedia e Traumatologia, Divisão de Doenças Infecciosas, São Paulo/, SP, Brazil

**Keywords:** Knee, Septic Arthritis, Epidemiology, *S. aureus*

## Abstract

**OBJECTIVES:**

To clinically and epidemiologically characterize a population diagnosed with and treated for septic arthritis of the knee, to evaluate the treatment results and to analyze the differences between patients with positive and negative culture results, patients with Gram-positive and Gram-negative bacterial isolates and patients with *S. aureus*- and non*-S. aureus*-related infections.

**METHODS:**

One hundred and five patients with septic knee arthritis were included in this study. The clinical and epidemiological data were evaluated. Statistical analysis was performed to compare patients with and without an isolated causative agent, patients with Gram-positive and Gram-negative pathogens and patients with *S. aureus*-related and non *S. aureus*-related infections.

**RESULTS:**

Causative agents were isolated in 81 patients. Gram-positive bacteria were isolated in 65 patients and Gram-negative bacteria were isolated in 16 patients. The most commonly isolated bacterium was *S. aureus*. Comparing cases with an isolated pathogen to cases without an isolated pathogen, no differences between the studied variables were found except for the longer hospital stays of patients in whom an etiological agent was identified. When comparing Gram-positive bacteria with Gram-negative bacteria, patients with Gram-positive-related infections exhibited higher leukocyte counts. Patients with *S. aureus-*related infections were more frequently associated with healthcare-related environmental encounters.

**CONCLUSION:**

*S. aureus* is the most common pathogen of septic knee arthritis. Major differences were not observed between infections with isolated and non-isolated pathogens and between infections with Gram-positive and Gram-negative bacteria. *S. aureus* infections were more likely to be associated with a prior healthcare environment exposure.

## INTRODUCTION

Although septic arthritis is a relatively uncommon orthopedic/rheumatologic disease, it is extremely important due to the severe consequences of non-optimal treatment 1. The age groups affected range from newborns to older adults, and the yearly incidence varies from 2 to 10 per 100,000 patients in the general population 1. In adults, the knee is the most affected site.

Despite the advances in drainage and antibiotic therapy techniques, complications including osteomyelitis, bone erosion, joint stiffness, fibrous ankylosis, sepsis and death may occur. In a recent series, complications were reported in 10% of cases; however, some authors have reported joint damage in 33% of cases and death in 11% of patients 2-4.

Treatment consists of early antibiotic therapy and the removal of purulent material by surgical drainage or needle aspiration, recognizing that controversy persists regarding which method is the most effective. The most common factors limiting successful treatment are the lack of suspicion for an infectious etiology in the initial symptomatic phases, delayed aspiration of synovial fluid and failed joint drainage 4-6.

With infectious agents resistant to multiple antibiotics increasingly reported from many centers, as well as the increased frequency of microorganisms not previously associated with septic arthritis, knowledge of the epidemiological characteristics of populations in each region is fundamental for adequate therapeutic planning. Notwithstanding, Gram-positive bacteria, particularly *Staphylococcus aureus*, are the most common infectious agents reported worldwide, accounting for more than 90% of cases in some series 3,5,7.

The aims of this study were to clinically and epidemiologically characterize the population diagnosed with knee septic arthritis and treated at our orthopedic hospital between 2006 and 2014 and to evaluate the treatment results. We also evaluated the differences between patients with positive and negative culture results, the differences between patients with isolated Gram-positive and Gram-negative bacteria and the differences between patients infected by *S. aureus*, the most common causative agent, and those with non*-S. aureus*-related infections.

## MATERIALS AND METHODS

This was a retrospective study of patients with a diagnosis of knee septic arthritis admitted to our hospital from 2006 to 2014. The study was approved by the ethics committee of our institution, and informed consent was obtained.

In this study, we included patients diagnosed with septic arthritis based on clinical findings and/or the presence of purulent material in the joint space and/or the isolation of a bacterial pathogen from the joint fluid/synovial membrane. Cases considered post-surgical infections, occurring less than one year after any knee surgical procedure, were excluded.

After inclusion, the following data were gathered from the medical records: sex, age, cause of infection (the routes classified as hematogenous, direct inoculation, i.e., following a previous joint injection, or contiguity based on the presence of local infection around the knee joint), origin of the infection allowing characterization as community-associated or healthcare-associated according to the CDC classification criteria 8, fever before hospital encounter, leukocyte count (with leukocytosis defined as a leukocyte count greater than 11,000 mm3), serum C-reactive protein (CRP) and erythrocyte sedimentation rate (ESR) values at the initial evaluation, synovial fluid Gram staining and culture results, number of surgical drainages, comorbidities (including immunosuppressive conditions), Charlson comorbidity index (CCI), time elapsed between the initial symptoms and surgical drainage, previous joint disease, previous surgery on the affected knee, previous surgery on the affected limb, systemic and joint complications and length of hospital stay.

All culture samples were collected in the surgical suite after extensive debridement of the joint. Samples were sent to the microbiology laboratory in bottles containing thioglycolate growth medium. Susceptibility tests were performed in most cases using the Vitek-2^®^ system (BioMérieux, France); when required, minimum inhibitory concentrations were obtained using the “e-test” and reported in accordance with the Clinical and Laboratory Standards Institute (CLSI) criteria in effect at that time.

Statistical analysis was performed to compare those patients with and without an isolated causative agent, with Gram-positive and Gram-negative agents, and those with *S. aureus*-related and non-*S. aureus*-related infections. We used the SPSS 20 for the statistical analysis. The chi-square test was used to analyze the associations of categorical data, the Mann-Whitney U -test was used for non-parametric testing of dependent variables and t-test was used for parametric testing of dependent variables. The tests were conducted with a 5% significance level.

## RESULTS

We evaluated 105 patients with a diagnosis of septic arthritis of the knee: 67 (63.8%) were male and 38 (36.2%) were female. The average age was 42.5 +/- 21.4 years (ranging from 1 to 92 years). Among the studied patients, 87 (82.9%) had hematogenous infections, 14 (13.3%) had direct inoculation infections and 4 (3.8%) were infected due to soft tissue infection around the knee (contiguity). Fever before admission was present in 39% of the included patients.

Leukocytosis was observed in 48 (45.7%) patients. The mean leukocyte count was 11,520 +/- 663. The mean CRP was 117.8 +/- 10.4, and the mean ESR was 53.3 +/- 2.9. All patients presented with elevated CRP and all but two (1.9%) patients presented with abnormal ESR upon hospital admission.

**Figure 1:**
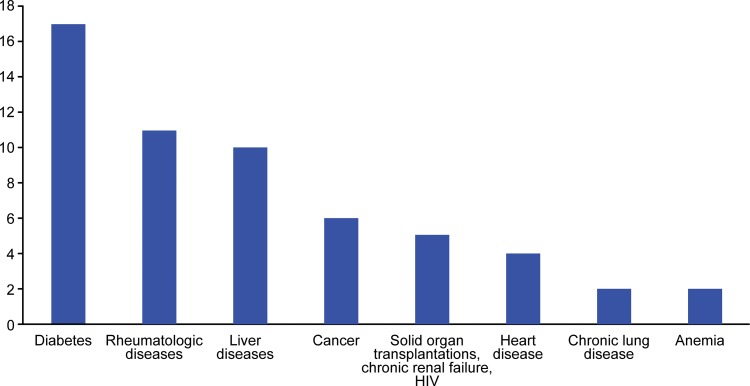
Most common comorbidities found in patients with septic knee arthritis.

Comorbidities were observed in 60 (57.1%) patients: the most common was diabetes (17 patients), followed by rheumatologic disease (11 patients), liver disease (10 patients), cancer (6 patients) and chronic renal failure, previous solid organ transplantation and HIV (5 patients each) (Figure 1). The mean number of comorbidities per patient was 1.02 +/- 0.12 (ranging from 0 to 7). Thirty patients (28.6%) were considered immunosuppressed. The mean CCI was 1.04 +/- 1.58.

The mean time between symptom onset and septic arthritis diagnosis was 10.9 +/- 1.6 days. Eleven (10.4%) patients experienced symptoms for more than 30 days before hospital admission. Thirty-six (34.3%) patients had knee joint disease before diagnosis, 14 (13.3%) patients had a previous knee surgery and 19 (18.0%) patients had a previous surgery in the affected limb.

All patients underwent open surgical drainage with access to the knee joint via the medial parapatellar route immediately after confirmation of the diagnosis. Eighty-three patients underwent only one surgical procedure, whereas 22 required more than one drainage procedure. The average number of procedures was 1.4 +/- 0.1.

Causative agents were isolated in 81 (77.1%) patients, whereas in 24 (22.8%) patients, no pathogen was isolated. Gram-positive bacteria were isolated in 65 (61.9%) patients and Gram-negative bacteria in 16 (15.2%) patients. The most common isolated bacterium was *S. aureus* (54 cases, 51.4%), including 13 methicillin-resistant isolates, followed by *Escherichia coli* (6 patients, 5.7%) and *Streptococcus pyogenes* (5 patients, 4.7%). No cases of mycobacterial or fungal infections were observed in the analyzed patients.

Regarding complications, 6 (5.7%) patients had septic shock and 2 (1.9%) of these patients died after intensive care treatment. One of these patients underwent a transfemoral amputation during the hospital stay. Twelve (11.4%) patients had local knee complications, 4 developed arthrosis, 5 developed osteomyelitis, 2 sustained severe loss of range of motion and 1 underwent knee disarticulation due to uncontrolled infection. The mean hospitalization period was 19.2 +/- 13.7 days.

Comparing cases with an isolated pathogen to those cases without an isolated pathogen, no differences between the studied variables were found except for longer hospital stays among the cases with an isolated pathogen (17.5 *versus* 14.3 days, respectively, *p*=0.012) (Table 1).

Comparing Gram-positive bacteria with Gram-negative bacteria, patients with Gram-positive-related infections demonstrated greater leukocytosis (12,631 *versus* 8,037, *p*=0.004) and tended to have higher CRP values (129.7 *versus* 84.9, *p*=0.098); however, the latter finding was not significant (Table 2).

More cases considered hospital-related (*p*=0.039) were observed among patients with *S. aureus-*related infections; moreover, they tended to have higher CRP values at their initial presentation (*p*=0.056) and exhibited less time from symptom onset to surgical drainage (*p*=0.091) compared with patients having non-*S. aureus*-related infections (Table 3).

## DISCUSSION

Although septic knee arthritis is an infrequently encountered pathologic condition worldwide, we observed a mean of 11.6 cases/year at our hospital during the studied period. Considering that we excluded surgical site infections and evaluated only the knee joint, the most common site for septic arthritis in adults, this incidence observed is higher than in most other multinational series 1,7.

The most common agent was *S. aureus*, similar to most previous series, and the majority of patients were infected by Gram-positive bacteria. The incidence of Gram-negative bacterial infections was approximately 20%, as previously described in the literature 9. In contrast to the study performed by Madruga Dias et al. 10, wherein the pathogen was not isolated in the majority of the cases, we were unable to isolate the causative agent in only 22.8% of patients, similar to the literature incidence 11. Some studies required a pathogen to be isolated by culture methods for consideration as a positive case, which explains the absence of these data in some series.

Whereas the studied patients ranged widely in age, the most commonly affected group was aged approximately 40 years, signifying that this disease is not only important in children and the elderly but also in other age groups. The mean age in this study was lower than that observed in the studies performed by Eberst-Ledoux et al. 9 and Madruga Dias et al. 10, similar to the study performed by Helito et al. 2 and higher than in the study by Miyahara et al. 12. However, in all these studies, adults were more frequently infected than children.

The main cause of infection was hematogenous spread. Whereas the incidence of knee infection following intraarticular injections is increasing, this etiology corresponded to only 13.3% of the cases 13.

Septic arthritis was associated with kidney disease, liver disease, cancer, diabetes, HIV infection and rheumatologic conditions. These pathologies are known to have common associations with joint infection 14. Most of the patients had at least one comorbidity and due to our hospital’s characteristics, a complex treatment center for cancer, HIV infection, transplantation and rheumatologic pathologies, these comorbidities were significant for lowering patient immunity. Similar to Khan et al. 15, diabetes was the most common comorbid factor in our study, whereas Madruga Dias et al. 10 observed pharmacological suppression as the most important factor.

Complications were less frequent than in other series. Both of the 2 (1.9%) deaths occurred in elderly patients with multiple complications. The mortality reported by Eberst-Ledoux et al. 9 was 5% and it was approximately 11% in the study of Coakley et al. 16. Kaandrop et al. 17 reported joint damage after septic arthritis affecting approximately 40% of patients, whereas we found local knee complications in 11.4% of patients. Delayed diagnosis, advanced age, underlying joint diseases and the presence of synthetic material within the joint are conditions associated with a poor prognosis. Delay in treatment for as little as 7 days might also result in poor outcomes 18.

We compared patients with and without an isolated pathogen. The only significant difference we found was that patients in whom a causative agent was isolated experienced longer hospital stays. Eberst-Ledoux et al. 9 found that patients with isolated bacteria were older, had at least one risk factor and had a higher mortality rate. Our study corroborates previous data reported by Madruga Dias et al. 10. In their series, patients with isolated bacteria had more risk factors for infection and longer hospital stays. The latter finding is likely due to more comorbidities, which lead to greater infection management difficulty.

Except for the higher leukocyte count and the higher CRP tendency, we could not identify any significant difference between those infections related to Gram-positive and those involving Gram-negative bacteria. When managing antibiotics, clinical or epidemiological clues may be useful for antibiotic targeting of the most probable pathogen. According to Goldenberg et al. 6, Gram-negative bacteria usually affect the very young, the very old, patients with a previous history of intravenous drug abuse and immunocompromised patients; however, we could not confirm these data in our study.

Because *S. aureus* is the most common septic arthritis pathogen, we searched for differences between the *S. aureus* and non-*S. aureus* infections. The only analyzed parameter that differed significantly was the patient history in that a history of a health service-related environmental encounter was associated with infection due to *S. aureus.* The growing incidence of methicillin-resistant *S. aureus* likely explains these findings 19. Some authors have suggested that in patients relating a health service environmental exposure, vancomycin or teicoplanin should be considered the first-choice empirical antibiotics 19. Patients with *S. aureus*-related infections also tended to have higher CRP values and shorter intervals between symptom onset and the surgical procedure.

*S. aureus* is the most common pathogen of septic knee arthritis. Major clinical and epidemiological markers for specific etiologic groups were not identified, with the exception of infections caused by *S. aureus* being more often associated with a patient history of a health service-related environmental.

## AUTHOR CONTRIBUTIONS

Helito CP was responsible for the creation of the research protocol, data analysis and manuscript writing. Teixeira PR was responsible for data gathering. Oliveira PR and Carvalho VC were responsible for data analysis. Pécora JR was responsible for study review. Camanho GL and Lima AL were responsible for coordination and study review. Demange MK was responsible for data analysis and study review.

## Figures and Tables

**Table 1:** Statistical analysis performed to compare patients with septic knee arthritis with and without an isolated causative agent.

	Isolated agent	Non-isolated agent	*p*
Number of patients	81 (77.1%)	24 (22.9%)	
Female sex	32 (39.5%)	6 (25%)	0.145
Age (average years)	42.5 +/- 19.8	42.6 +/- 26.5	0.2
Cause of infection			0.527
Hematogenous	66 (81.5%)	21 (87.5%)	
Direct inoculation	11 (13.6%)	3 (12.5%)	
Contiguity	4 (4.9%)	0	
History			0.659
Community	57 (70.4%)	18 (75.0%)	
Healthcare-related	24 (29.6%)	6 (25.0%)	
Fever (number of patients)	30 (37.0%)	11 (45.8%)	0.438
Leukocyte count	11808 +/- 7450	10560 +/- 3590	0.76
CRP level	122.1 +/- 114.5	103.2 +/- 70.8	0.882
ESR	53.8 +/- 31.6	51.6 +/- 23.4	0.743
Number of surgical procedures	1.5 +/- 1.1	1.2 +/- 0.6	0.185
Comorbidities (number of patients)	47 (58.0%)	13 (54.2%)	0.737
Immunosuppression (number of patients)	25 (30.9%)	5 (20.8%)	0.339
Charlson comorbidity index (mean)	1.0 +/- 1.6	0.8 +/- 1.3	0.54
Time to surgery (days)	9.6 +/- 13.4	15.5 +/- 26.3	0.552
Previous joint disease	30 (30.0%)	6 (25.0%)	0.275
Previous knee surgery	12 (14.8%)	2 (8.3%)	0.412
Previous limb surgery	17 (21.0%)	2 (8.3%)	0.157
Systemic complications	6 (7.4%)	0	0.17
Joint complications	10 (12.45)	1 (4.2%)	0.25
Length of hospital stay (days)	20.7 +/- 14.6	14.3 +/- 9.2	0.012

CRP – C-reactive protein; ESR – erythrocyte sedimentation rate.

**Table 2:** Statistical analysis performed to compare patients with septic knee arthritis with Gram-positive and Gram-negative pathogens.

	Gram -	Gram +	*p*
Number of patients	16 (19.8%)	65 (80.2%)	
Female sex	9 (56.2%)	23 (35.4%)	0.126
Age (average years)	44.1 +/- 21.0	42.1 +/- 19.7	0.714
Cause of infection			0.502
Hematogenous	13 (81.3%)	53 (81.5%)	
Direct inoculation	3 (18.7%)	8 (12.3%)	
Contiguity	0	4 (6.2%)	
History			0.287
Community	13 (81.3%)	44 (67.7%)	
Healthcare-related	3 (18.7%)	21 (32.3%)	
Fever (number of patients)	5 (31.3%)	25 (38.5%)	0.593
Leukocyte count	8037 +/ 4940	12631 +/- 7708	0.004
CRP level	84.9 +/- 81.5	129.7 +/- 120.2	0.098
ESR	44.6 +/- 24.2	55.3 +/- 33.0	0.383
Number of surgical procedures	1.5 +/- 0.9	1.5 +/- 1.1	0.808
Comorbidities (number of patients)	9 (56.3%)	38 (58.5%)	0.872
Immunosuppression (number of patients)	6 (37.5%)	19 (29.2%)	0.521
Charlson comorbidity index (mean)	1.1 +/- 1.4	1.0 +/- 1.7	0.94
Time to surgery (days)	14.5 +/- 22.1	8.3 +/- 10.0	0.133
Previous joint disease	6 (37.5%)	24 (36.9%)	0.966
Previous knee surgery	4 (25.0%)	8 (12.3%)	0.2
Previous limb surgery	5 (31.3%)	12 (18.5%)	0.26
Systemic complications	0	6 (9.2%)	0.593
Joint complications	4 (25.0%)	6 (9.2%)	0.086
Length of hospital stay (days)	20.6 +/- 15.7	20.7 +/- 14.4	0.472

CRP – C-reactive protein; ESR – erythrocyte sedimentation rate.

**Table 3:** Statistical analysis performed to compare patients with septic knee arthritis with *S. aureus*-related infections *versus* non-*S. aureus*-related infections.

	Other bacteria	*S. aureus*	*p*
Number of patients	27 (33.3%)	54 (66.7%)	
Female sex	13 (48.1%)	19 (35.1%)	0.261
Age (average years)	43 +/- 23.5	42.3 +/- 19.5	0.2
Cause of infection			0.502
Hematogenous	22 (81.5%)	44 (81.5%)	
Direct inoculation	5 (18.5%)	6 (11.1%)	
Contiguity	0	4 (7.4%)	
History			0.039
Community	23 (85.2%)	34 (63.0%)	
Healthcare-related	4 (14.8%)	20 (37.0%)	
Fever (number of patients)	10 (37.0%)	20 (37.0%)	1
Leukocyte count	11027 +/- 5137	12184 +/- 7989	0.378
CRP level	89.8 +/- 72.3	137.7 +/- 127.1	0.056
ESR	55.1 +/- 26.7	53.2 +/- 32.8	0.752
Number of surgical procedures	1.7 +/- 1.2	1.4 +/- 0.8	0.631
Comorbidities (number of patients)	13 (48.2%)	34 (63.0%)	0.203
Immunosuppression (number of patients)	8 (29.6%)	17 (31.5%)	0.865
Charlson comorbidity index (mean)	0.9 +/- 1.5	1.1 +/- 1.6	0.66
Time to surgery (days)	12.1 +/- 22.0	8.3 +/- 10.5	0.091
Previous joint disease	9 (33.3%)	21 (38.9%)	0.625
Previous knee surgery	5 (18.5%)	7 (13.0%)	0.507
Previous limb surgery	6 (22.2%)	11 (20.4%)	0.847
Systemic complications	1 (3.7%)	5 (9.3%)	0.658
Joint complications	5 (18.5%)	5 (9.3%)	0.232
Length of hospital stay (days)	19.6 +/- 12.2	21.3 +/- 14.8	0.313

CRP – C-reactive protein; ESR – erythrocyte sedimentation rate.
